# Modeling Sporadic Alzheimer's Disease in Human Brain Organoids under Serum Exposure

**DOI:** 10.1002/advs.202101462

**Published:** 2021-08-02

**Authors:** Xianwei Chen, Guoqiang Sun, E Tian, Mingzi Zhang, Hayk Davtyan, Thomas G. Beach, Eric M. Reiman, Mathew Blurton‐Jones, David M. Holtzman, Yanhong Shi

**Affiliations:** ^1^ Division of Stem Cell Biology Research Department of Developmental and Stem Cell Biology Beckman Research Institute of City of Hope 1500 E. Duarte Rd Duarte CA 91010 USA; ^2^ Institute for Memory Impairments & Neurological Disorders and Sue & Bill Gross Stem Cell Research Center University of California Irvine Irvine CA 92697 USA; ^3^ Banner Sun Health Research Institute 105015 West Santa Fe Drive Sun City AZ 85351 USA; ^4^ Banner Alzheimer Institute 901 East Willetta Street Phoenix AZ 95006 USA; ^5^ Department of Neurobiology & Behavior Institute for Memory Impairments & Neurological Disorders and Sue & Bill Gross Stem Cell Research Center University of California Irvine Irvine CA 92697 USA; ^6^ Department of Neurology Hope Center for Neurological Disorders Knight Alzheimer's Disease Research Center Washington University in St. Louis St. Louis MO 63110 USA

**Keywords:** brain organoids, disease modeling, induced pluripotent stem cells, serum exposure, sporadic Alzheimer's disease

## Abstract

Alzheimer's disease (AD) is a progressive neurodegenerative disease with no cure. Huge efforts have been made to develop anti‐AD drugs in the past decades. However, all drug development programs for disease‐modifying therapies have failed. Possible reasons for the high failure rate include incomplete understanding of complex pathophysiology of AD, especially sporadic AD (sAD), and species difference between humans and animal models used in preclinical studies. In this study, sAD is modeled using human induced pluripotent stem cell (hiPSC)‐derived 3D brain organoids. Because the blood–brain barrier (BBB) leakage is a well‐known risk factor for AD, brain organoids are exposed to human serum to mimic the serum exposure consequence of BBB breakdown in AD patient brains. The serum‐exposed brain organoids are able to recapitulate AD‐like pathologies, including increased amyloid beta (A*β*) aggregates and phosphorylated microtubule‐associated tau protein (p‐Tau) level, synaptic loss, and impaired neural network. Serum exposure increases A*β* and p‐Tau levels through inducing beta‐secretase 1 (BACE) and glycogen synthase kinase‐3 alpha / beta (GSK3*α*/*β*) levels, respectively. In addition, single‐cell transcriptomic analysis of brain organoids reveals that serum exposure reduced synaptic function in both neurons and astrocytes and induced immune response in astrocytes. The human brain organoid‐based sAD model established in this study can provide a powerful platform for both mechanistic study and therapeutic development in the future.

## Graphical abstract

Brain organoids are exposed to human serum to mimic the serum exposure consequence of the blood–brain barrier (BBB) breakdown in Alzheimer's disease (AD) patient brains, which recapitulates several AD‐like pathologies, including increased amyloid beta (A*β*) and phosphorylated microtubule‐associated tau protein (p‐Tau) level, synaptic loss, and impaired neural network. This human brain organoid‐based sporadic AD (sAD) model provides a powerful platform for both mechanistic study and therapeutic development in the future.

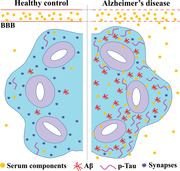

## 1 Introduction

Alzheimer's disease (AD) is a progressive neurodegenerative disease with no cure, characterized by extracellular amyloid beta (A*β*) plaques, intracellular phosphorylated microtubule‐associated tau protein (p‐Tau) tangles, and age‐related memory loss. Over 50 million people worldwide are believed to live with AD or other dementia, and 5.8 million Americans suffer from AD in 2019.^[^ ^1^ ^]^ AD can be classified into familial AD (fAD) and sporadic AD (sAD), which accounts for less than 5% and more than 95% of AD cases, respectively.^[^ ^2^ ^]^ A large number of attempts have been made to develop anti‐AD drugs in the past decades. However, all drug development programs for disease‐modifying therapies have failed.^[^ ^2d^ ^]^ Possible reasons for the high failure rate in AD drug development include late intervention, incomplete understanding of complex pathophysiology of AD (especially sAD), amyloid only‐based therapeutics, and species difference between humans and animal models used in preclinical studies.^[^ ^2^, ^3^ ^]^ Therefore, developing human cellular models for AD, especially sAD, and identifying targets beyond A*β* may lead to more effective therapeutic development.

The induced pluripotent stem cell (iPSC) technology has transformed the fields of stem cell biology and regenerative medicine.^[^ ^4^ ^]^ The human induced pluripotent stem cells (hiPSCs) have been used for modeling a variety of human diseases because of their human origin, easy accessibility, and ability to give rise to disease‐relevant cell types.^[^ ^5^ ^]^ iPSC‐based disease modeling is well‐suited for studying early‐onset disorders,^[^ ^6^ ^]^ but can be more challenging for late‐onset diseases because iPSCs are considered phenotypically young.^[^ ^7^ ^]^ This challenge can be overcome by inducing cellular aging or introducing aging‐related events to aid in successful modeling of late‐onset diseases.^[^ ^8^ ^]^

hiPSC‐derived 3D organoids have been developed for a variety of applications due to their resemblance to endogenous cell organization and organ structure, and are particularly useful because they allow us to study disease phenotypes in a cellular context that mimics human physiology and development.^[^ ^9^ ^]^ Recently, brain organoids derived from hiPSCs have been widely used for modeling human neurological disorders,^[^ ^10^ ^]^ including AD.^[^ ^11^ ^]^ While organoid models provide comprehensive tools for iPSC‐based disease modeling, it is not without limitations. For example, the lack of blood stream in the current organoid system prevents us from modeling the effects resulted from serum exposure resulted from the blood–brain barrier (BBB) leakage, a well‐known risk factor for AD, which can be caused by aging,^[^ ^12^ ^]^ the greatest risk factor for AD, and apolipoprotein E4 (*ApoE4*),^[^ ^13^ ^]^ a well identified AD risk gene.^[^ ^2^, ^14^ ^]^

Because the etiology of sAD is complex and remains largely unclear, it has been challenging to model sAD. In this study, we have modeled sAD using human iPSC‐derived brain organoids exposed to serum to mimic serum exposure resulted from BBB breakdown that has been observed in aging human brains and considered a key factor in AD pathogenesis.^[^ ^12^, ^15^ ^]^ We found that serum‐exposed brain organoids were able to recapitulate multiple aspects of AD‐like pathologies. Single‐cell transcriptomic analysis of brain organoids revealed that serum exposure caused reduced synaptic function in both neurons and astrocytes and induced immune response in astrocytes.

## 2 Results

### 2.1 Generation and Characterization of hiPSC‐Derived Brain Organoids

**Figure 1:**
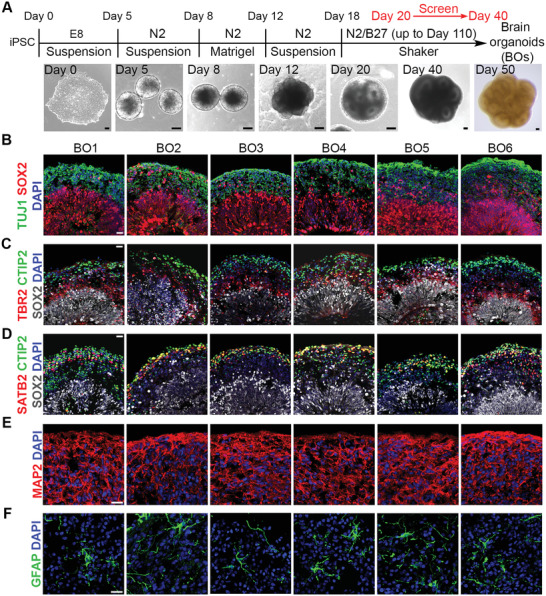
Generation and characterization of hiPSCs‐derived brain organoids. A) Schematic illustration of brain organoid protocol and representative phase images at different stages. Scale bar, 100 µm. B) Immunostaining of the neural progenitor marker SOX2 and the neuronal marker TUJ1 in brain organoids at day 50–60. C) Immunostaining of SOX2, the intermediate progenitor marker TBR2, and the cortical deep layer marker CTIP2 in brain organoids at day 50–60. D) Images of immunostaining of SOX2, CTIP2, and the cortical upper layer marker SATB2 in brain organoids at day 50–60. E) Immunostaining of the neuronal marker MAP2 in brain organoids at day 90. F) Immunostaining of the astrocyte marker GFAP in brain organoids at day 110. B–F) Scale bar, 20 µm.

In order to model sAD using human cells, brain organoids were generated from hiPSCs following our published protocol^[^ ^10e^ ^]^ adapted from that described by Lancaster et al.^[^ ^9^, ^10^ ^]^ and Qian et al.^[^ ^10b^ ^]^ with modifications (**Figure** **1**A). In order to generate brain organoids with good reproducibility and homogeneity, we developed a screening procedure to select organoids during day 20 to day 40 of organoid development (Figure 1A). Only brain organoids that were composed of high‐quality neural rosettes showed bright cycles by phase‐contrast microscopy (Figure 1A, day 20, day 40) were kept for further experiments. At day 50 to day 60, the SOX2‐positive ventricular zone‐like (VZ) layer and the TUJ1+ neuronal layer were observed in all lines of brain organoids (Figure 1B).

Since the cortex is one region that is mainly affected in AD patient brains,^[^ ^16^ ^]^ brain organoids containing the cortical region were used to model sAD in this study. Further characterization of day 50–60 brain organoids revealed that these organoids contained a TBR2^+^ subventricular zone‐like (SVZ) layer between the SOX2^+^ VZ layer and the cortical deep layer marker CTIP2^+^ cortical plate‐like (CP) layers (Figure 1C). In addition, the brain organoids contained the cortical upper layer marker SATB^+^ neurons that were partially overlapped with the CTIP2^+^ neurons (Figure 1D), consistent with published reports^[^ ^10^, ^17^ ^]^. These results indicate that the brain organoids we generated mimic the cortical region, a brain region that is highly affected in AD. Moreover, we were able to detect both MAP2^+^ neurons and GFAP^+^ astrocytes in all brain organoids (Figure 1E,F). Together, the brain organoids generated in this study were derived from human cells, exhibited good reproducibility, displayed the cortex‐like structure, contained AD pathology‐relevant cell types, such as neurons and astrocytes, therefore are highly suitable for modeling sAD.

### 2.2 Serum Exposure Induces A*β*‐Like  Pathology through Increasing Beta‐Secretase 1(BACE) Abundance

**Figure 2:**
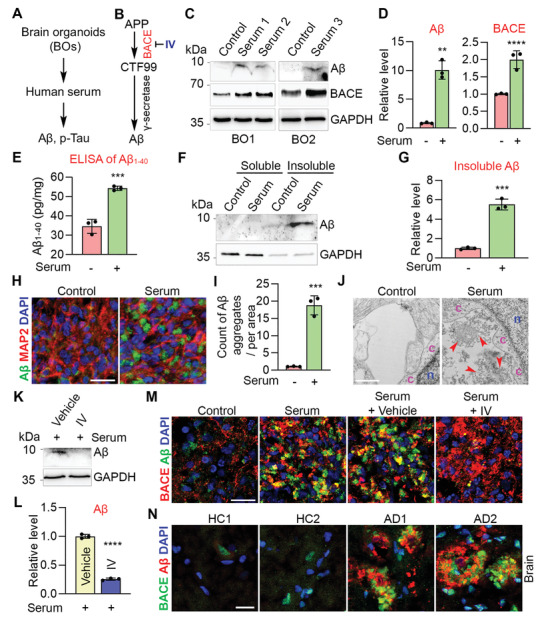
Serum exposure induces A*β*‐like pathology through increasing BACE abundance. A) Schematics of modeling AD pathologies using BOs. B) Schematics of amyloidogenic APP processing pathway. IV: BACE inhibitor. C) Western blot of A*β* (D54D2), BACE and GAPDH in BOs treated without (control) or with serum. Each lyaste was from pooled 3–5 individual BOs. D) Quantification of A*β* (D54D2) and BACE levels and normalized to GAPDH. *n* = three experiments in BO1 and BO2. E) ELISA of A*β*_1‐40_ in control and serum‐treated BO6. Each lysate was from pooled 5–10 individual BOs. *n* = three experimental repeats. F) Western blot of soluble and insoluble A*β* (D54D2) in control and serum‐treated BO1. Each lysate was from pooled 7–10 individual BOs. G) Quantification of insoluble A*β* and normalized to GAPDH. *n* = 3 quantitative repeats. H) Immunostaining of A*β* (D54D2) and MAP2 in control and serum‐treated BO1. I) Counts of A*β* aggregates in (H). *n* = 3 images from three individual BOs. J) Transmission electron microscopy (TEM) of control and serum‐treated BO2. n: nucleus, c: cytoplasm; arrowhead: extracellular aggregate. K) Western blot of A*β* (D54D2) and GAPDH in BO2 treated with vehicle, or inhibitor IV under serum exposure. Each lysate was from pooled 6–7 individual BOs. L) Quantification of A*β* level in (K) and normalized to GAPDH. *n* = 3 quantitative repeats. M) Immunostaining of A*β* (D54D2) and BACE in BO1 of control, serum‐treated, and serum plus vehicle or inhibitor IV treatment. N) Immunostaining for A*β* (6E10) and BACE in cortex of AD patients and healthy controls (HC). D,E,G,I,L) Error bars are SD of the mean, **p < 0.01, ***p < 0.001 and ****p < 0.0001 by unpaired two‐tailed *t*‐test. Scale bar: 20 µm for panels (H, M, N); 1 µm for panel (J).

BBB leakage is a well‐known risk factor for sAD,^[^ ^13^, ^15^, ^18^ ^]^ which could be induced by aging^[^ ^12^ ^]^ or ApoE4,^[^ ^13^ ^]^ indicating that AD patient brains had been exposed to serum components resulted from BBB breakdown. We hypothesize that serum exposure could induce AD pathologies, such as A*β* plaques and increased p‐Tau level, in the brain. However, it is challenging to model the BBB leakage using current brain organoid platform because there is no blood stream in brain organoids. To meet the challenge, we exposed brain organoids to human serum to mimic serum exposure. To assess the effects of serum exposure on AD pathologies, brain organoids were treated with human serum for about 12 days, then analyzed for AD pathologies, including A*β* and p‐Tau levels (**Figure** **2**A). It is well known that A*β* is generated from amyloid precursor protein (APP) through sequential cleavages, first by BACE to generate beta C‐terminal fragments and then by *γ*‐secretase complex to generate A*β* (Figure 2B).^[^ ^19^ ^]^ As expected, serum treatment dramatically increased A*β* level in brain organoids, compared to control organoids without serum treatment (Figure 2C,D). Increase of A*β* level by serum exposure was confirmed by A*β* ELISA in serum‐treated brain organoids, compared to control organoids (Figure 2E). Next, we asked whether increased A*β* in serum‐treated organoids was insoluble. The soluble and insoluble fractions of A*β* were isolated from control and serum‐treated brain organoids. The majority of A*β* was detected in the insoluble fraction of serum‐treated brain organoids (Figure 2F,G). This result indicates that A*β* may form aggregates in serum‐treated brain organoids. To test this hypothesis, we first performed A*β* staining and showed that the deposit of A*β* aggregates was dramatically increased in serum‐treated brain organoids, compared to control organoids (Figure 2H,I). The extracellular aggregates were also observed in serum‐treated brain organoids using transmission electron microscopy (TEM) (Figure 2J). Together, these data demonstrate that serum exposure could induce A*β*‐like pathology in brain organoids.

Next, we explored mechanisms underlying serum exposure induced A*β* pathology. In contrast to the nonamyloidogenic pathway of APP processing, in the amyloidogenic pathway, APP is first cleaved by BACE (Figure 2B),^[^ ^19a^ ^]^ a key enzyme that drives the production of A*β*. We hypothesize that serum exposure could induce A*β* production through increasing BACE expression. Indeed, the abundance of BACE protein was significantly increased in serum‐treated brain organoids, compared to control organoids (Figure 2C,D). Moreover, we observed enriched BACE protein in A*β* aggregate areas in serum‐treated organoids (Figure 2M). Treatment of brain organoids with the BACE inhibitor IV (IV) reduced A*β* level substantially under serum exposure condition (Figure 2K–M). These results together indicate that serum exposure could enhance BACE expression to induce A*β* accumulation. Importantly, increased abundance of BACE protein was also observed around A*β* plaque areas in AD patient brains compared to healthy controls (Figure 2N). Taken together, the serum‐exposed brain organoid model allowed us to recapitulate A*β*‐like pathology and identify serum‐induced elevation of BACE expression as one of the mechanisms underlying A*β* pathology in AD.

### 2.3 Serum Exposure Induces Elevated p‐Tau Level in Brain Organoids through Glycogen Synthase Kinase‐3 Alpha / Beta (GSK3*α*/*β*)

**Figure 3:**
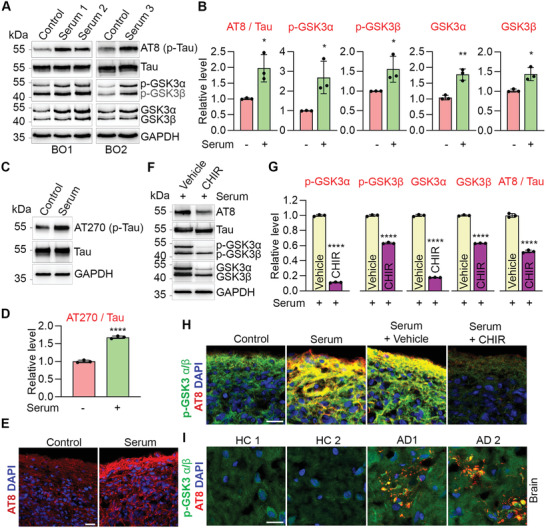
Serum exposure induces p‐Tau through GSK3*α*/*β*. A) Western blot of p‐Tau (AT8), Tau, p‐GSK3*α*/*β*, GSK3*α*/*β* and GAPDH in BOs treated without (control) or with serum. Each lyaste was from pooled 3–5 individual BOs. B) Quantification of p‐Tau (AT8), p‐GSK3*α*, p‐GSK3*β*, GSK3*α*, and GSK3*β* levels. p‐Tau was normalized to total Tau and the others were normalized to GAPDH. *n* = three experiments in BO1 and BO2. C) Western blot of p‐Tau (AT270), Tau and GAPDH in control and serum‐treated BO1. Each lyaste was from pooled 3–5 individual BOs. D) Quantification of p‐Tau (AT270) level and normalized to total Tau. *n* = 3 quantitative repeats. E) Immunostaining for p‐Tau (AT8) in control and serum‐treated BO2. F) Western blot of p‐Tau (AT8), Tau, p‐GSK3*α*/*β*, GSK3*α*/*β* and GAPDH in BO2 treated with vehicle, or GSK3*α*/*β* inhibitor CHIR99021 (CHIR) under serum exposure. G) Quantification of p‐GSK3*α*/*β*, GSK3*α*/*β* and p‐Tau (AT8) levels. p‐Tau (AT8) was normalized to total Tau and the others were normalized to GAPDH. *n* = 3 quantitative repeats. H) Immunostaining for p‐Tau (AT8) and p‐GSK3*α*/*β* in BO2 treated with vehicle control, serum, serum plus vehicle or serum plus CHIR treatment. I) Immunostaining for p‐Tau (AT8) and p‐GSK3*α*/*β* in cortex of AD patients and healthy controls (HC). E,H,I) Scale bar, 20 µm. B,D,G) Error bars are SD of the mean; *p < 0.05, **p < 0.01, and ****p < 0.0001 by unpaired two‐tailed *t*‐test.

Next, we assessed the effect of serum exposure on p‐Tau level using the AT8 antibody that recognizes phosphorylated Ser202 and Thr205 of Tau in brain organoids treated with or without serum. Serum exposure significantly increased p‐Tau, but not total Tau level in brain organoids (**Figure** **3**A,B). The increased level of p‐Tau was also observed using the AT270 antibody that recognizes phosphorylated Thr181 of Tau (Figure 3C,D). These results indicate that serum treatment could induce phosphorylation of Tau protein at multiple sites. Consistent with the Western blot result, increased p‐Tau level was also observed by immunostaining in serum‐treated brain organoids (Figure 3E). These results together indicate that serum exposure could induce elevated p‐Tau level in brain organoids.

Next, we explored mechanisms underlying serum exposure‐induced elevation of p‐Tau level. Several Tau protein kinases have been identified, including GSK3*α*/*β*.^[^ ^20^ ^]^ We asked whether serum exposure could induce p‐Tau pathology through enhancing the expression and/or activity of the Tau protein kinases. We found both phosphorylated GSK3*α*/*β* (Y216/Y279) (p‐GSK3*α*/*β*) and total GSK3*α*/*β* levels were increased substantially in serum‐treated brain organoids, compared to control organoids (Figure 3A,B). To confirm that serum exposure could induce p‐Tau level through GSK3*α*/*β*, brain organoids were treated with vehicle control or CHIR99021 (CHIR), an ATP‐competitive GSK3*α*/*β* inhibitor,^[^ ^21^ ^]^ under serum exposure condition. Both p‐GSK3*α*/*β* and total GSK3*α*/*β* levels were reduced substantially by CHIR in brain organoids, compared to vehicle control (Figure 3F,G). CHIR treatment reduced p‐Tau level in brain organoids under serum exposure condition (Figure 3F–H), indicating that serum exposure could induce p‐Tau level through enhancing GSK3*α*/*β* expression or activity. Together, these data indicate that serum exposure could induce p‐Tau level through enhancing its kinase GSK3*α*/*β* expression and / or activity. Increased levels of p‐GSK3*α*/*β* and p‐Tau were also detected in AD patient cortex tissues (Figure 3I). Taken together, this serum‐exposed brain organoid model allowed us to recapitulate the pathology of elevated p‐Tau and identify enhanced GSK3*α*/*β* expression and/or activity as one of the underlying mechanisms.

### 2.4 Serum Exposure Induces Synaptic Loss and Impairs Neural Network

**Figure 4:**
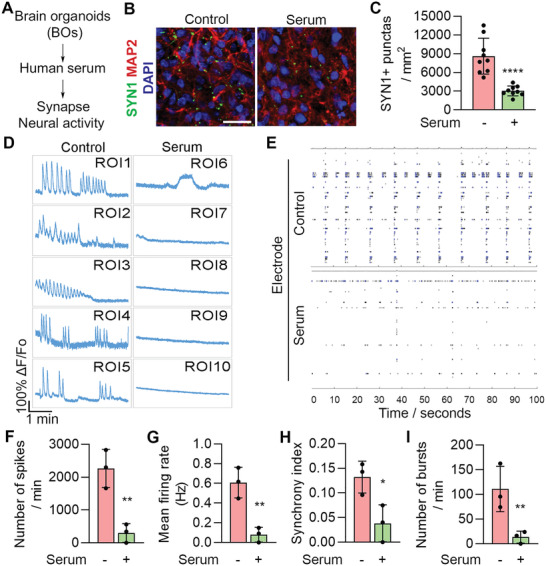
Serum exposure induces synaptic loss and reduces neural function. A) Schematics of modeling AD pathologies using brain organoids. B) Immunostaining for SYN1 and MAP2 in control and serum‐treated BO1. Scale bar, 20 µm. C) Quantification of SYN1+ synaptic punctas. *n* = 9 images from three individual BOs, with 3 sections per BO. D) Calcium imaging of BO4 treated without (control) or with serum for 7 days. The fluorescence intensity in five regions of interest (ROI) is shown. E) MEA analysis of BO2 treated without (control) or with serum for 7 days. Graphs illustrate MEA recording generated from the raw data of a spike raster plot, using the number of spikes recorded over 100 s. F–I) Quantification of the MEA parameters. *n* = 3 experimental repeats. C,F–I) Error bars are SD of the mean; *p < 0.05, **p < 0.01 and ****p < 0.0001 by unpaired two‐tailed *t*‐test.

Besides A*β* and p‐Tau pathologies, synaptic loss is another key feature of AD.^[^ ^22^ ^]^ We asked whether synaptic loss could be recapitulated in our brain organoid model. Brain organoids were treated with or without human serum and stained for synapsin‐1 (SYN1). The SYN1^+^ synaptic puncta were reduced substantially in serum‐treated brain organoids, compared to control organoids (**Figure** **4**A–C). These results indicate that serum exposure could induce synaptic loss, a key AD pathological feature, in brain organoids.

Next, we explored the effect of serum exposure on neural activity in brain organoids by calcium imaging. Unlike control organoids that exhibited frequent bursts of calcium surges, substantially reduced calcium signaling was detected in serum‐treated brain organoids (Figure 4D). In addition to calcium imaging, we evaluated neural network activity in control and serum‐treated brain organoids by microelectrode arrays (MEA). We were able to detect multiple synchronized bursts and spikes by MEA in control brain organoids (Figure 4E–I). In contrast, serum‐treated brain organoids displayed substantially reduced neural network activity, including reduced number of spikes, bursts, mean firing rate, and synchrony index (Figure 4E–I). These results together indicate that serum exposure could impair neural network activity in brain organoids.

### 2.5 Response of Brain Organoids to Compound Treatments under Serum Exposure

**Figure 5:**
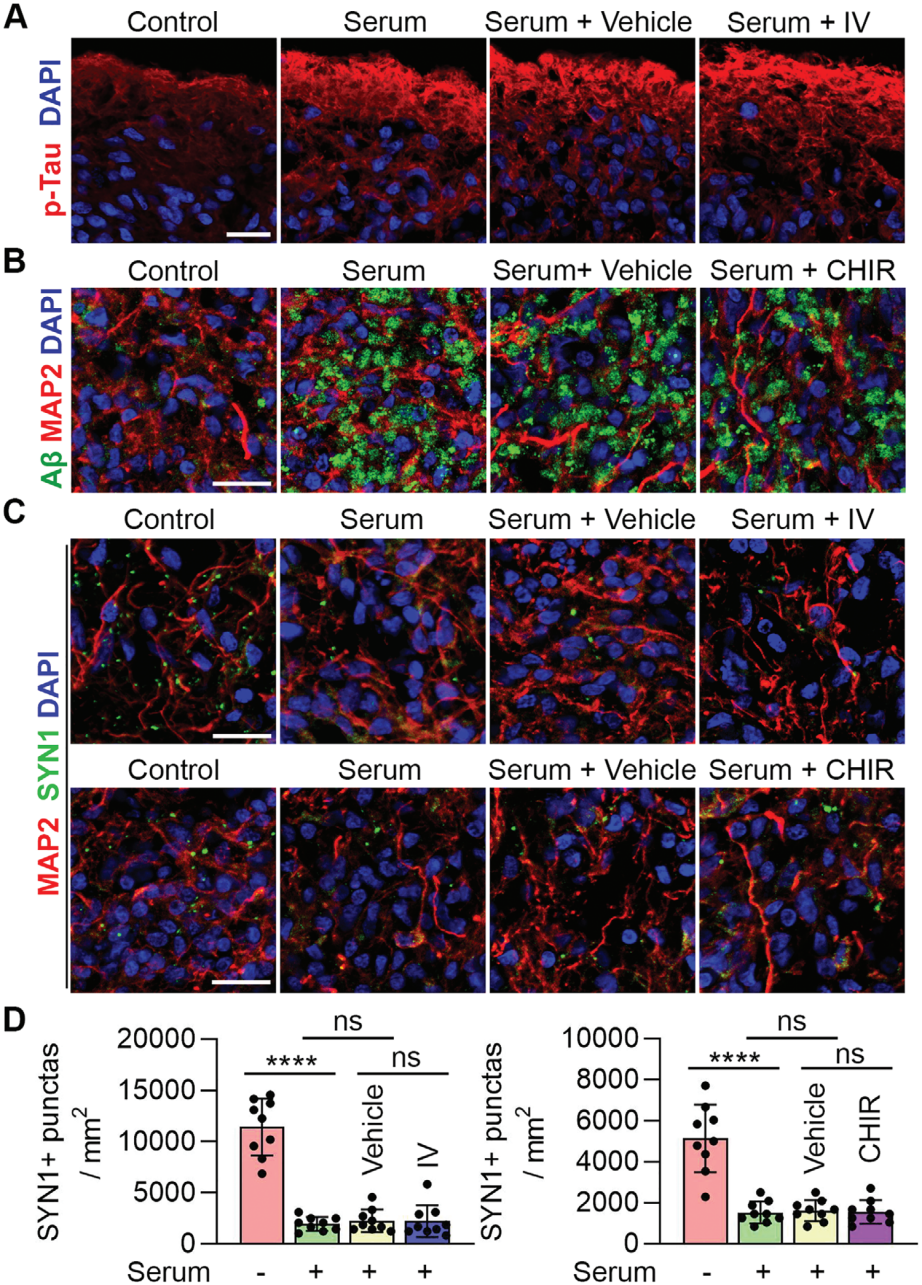
Response of brain organoids to compound treatment under serum exposure. A) Immunostaining for p‐Tau (AT8) in BO1 treated with control (no serum), serum, serum plus vehicle or serum plus inhibitor IV. B) Immunostaining for A*β* (D54D2) and MAP2 in BO5 treated with control (no serum), serum, serum plus vehicle or serum plus inhibitor CHIR. C) Immunostaining for SYN1 and MAP2 in BO1 (upper panel) and BO5 (lower panel) treated with control (no serum), serum, serum plus vehicle, serum plus inhibitor IV (upper panel) or serum plus CHIR (lower panel). D) Quantification of SYN1+ synaptic punctas. Error bars are SD of the mean; *n* = 9 images from three individual BOs, with 3 sections per BO. ns: not significant, ****p < 0.0001 by unpaired two‐tailed *t*‐test. A–C) Scale bars, 20 µm.

There is no cure for AD and treatments are symptomatic only. Therefore, there is an urgent need to develop effective therapies for AD. It has been shown that blocking APP processing could reduce p‐Tau level in fAD brain organoids,^[^ ^11e^ ^]^ we asked whether blocking APP processing could reduce p‐Tau level in our sAD model under serum exposure condition. Brain organoids were treated with vehicle control or BACE inhibitor IV under serum exposure condition. However, Inhibitor IV treatment failed to reduce p‐Tau level in compound‐treated organoids under serum exposure (**Figure** **5**A). This result suggests that serum exposure could induce p‐Tau level independently of A*β* level.

Next, we asked whether the A*β* pathology could be alleviated by reducing p‐Tau level. To answer this question, brain organoids were treated with GSK3*α*/*β* inhibitor CHIR under serum exposure condition to reduce p‐Tau level. However, CHIR treatment had no effect on A*β* level in serum‐exposed brain organoids (Figure 5B), suggesting that serum‐induced A*β* level may not be caused by the increase of p‐Tau level.

We then asked whether reducing A*β* or p‐Tau level could rescue synaptic loss in serum‐exposed brain organoids. Treatment with either BACE inhibitor IV or CHIR failed to increase SYN1^+^ synaptic puncta in serum‐exposed organoids (Figure 5C,D), suggesting that serum exposure could induce synaptic loss independently of A*β* and p‐Tau. These results together indicate that serum exposure could induce multiple AD pathologies in parallel, thus targeting multiple pathologies using combinatorial therapies may be necessary to slow down the progression of the disease.

### 2.6 Single Cell Transcriptomic Analysis Reveals Reduced Synaptic Function in Neurons of Serum‐Exposed Brain Organoids

**Figure 6:**
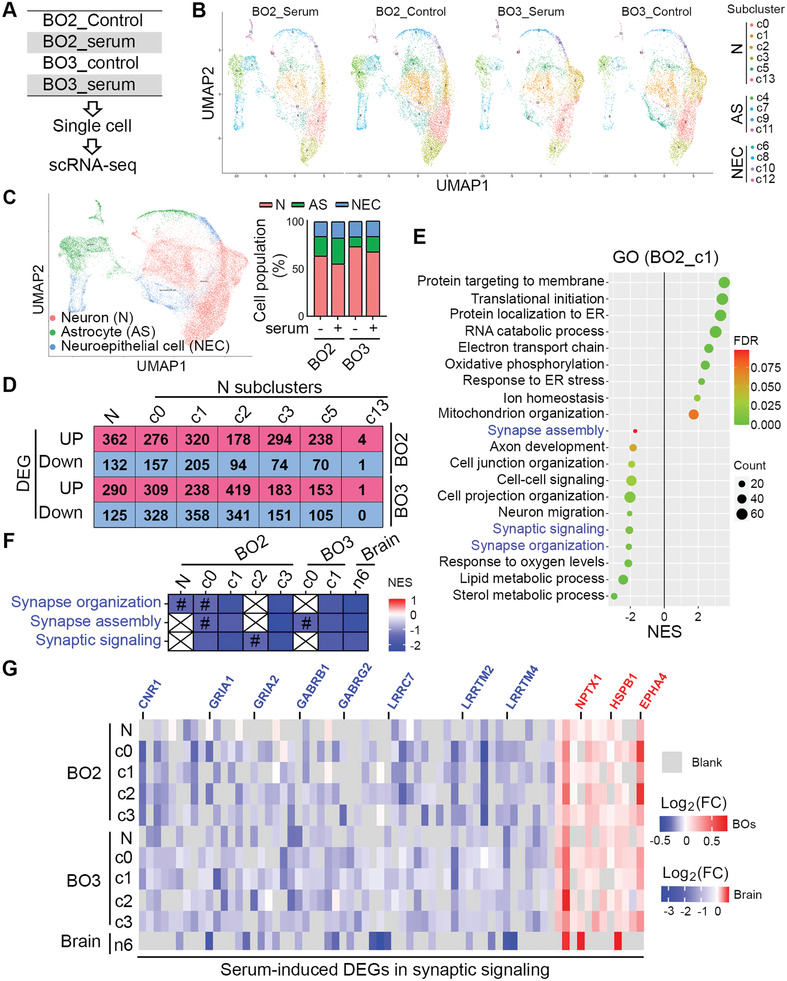
Serum exposure reduced synaptic function in neurons of brain organoids revealed by scRNA‐seq. A) Schematics of scRNA‐seq of BOs treated without (control) and with serum for 13–14 days. Single cells of each group were from pooled 3–5 individual BOs. B) UMAP visualization showing subcluster of neurons (N), astrocytes (AS) and neuroepithelial cells (NEC) in BO2 and BO3. C) UMAP visualization showing clustering of single cell colored by cell types. The composition of cells is shown in each sample. D) Number of differentially expressed genes (DEGs) in neurons (N) and neural subclusters that were up‐ or down‐regulated in serum‐treated BOs. E) GSEA result (Gene Ontology, GO) using DEGs of neural c1 subcluster in BO2. p‐Value < 0.05. F) Three GO categories related to synaptic function are shown in neuron and neural subclusters of BOs and AD cortex published by Grubman et al. Colored by NES value for each category. *p*‐value < 0.05 except labeled with #. #: *p*‐Value larger than 0.05 and less than 0.1. G) Neural DEGs involved in synaptic functions from both BOs (*p‐*value < 0.05) and AD cortex (FDR < 0.05) published by Grubman et al. Colored by Log2(FC) values. FC: fold change.

To investigate how serum exposure could have affected synaptic function and which cell type was involved during this process, we performed single cell RNA sequencing (scRNA‐seq) of control and serum‐treated brain organoids that were derived from two different hiPSC lines (BO2 and BO3) (**Figure** **6**A). The subcluster analysis uncovered 14 cell subclusters in both BO2 and BO3 of both control and serum‐treated brain organoids, including 6 neuronal subclusters, 4 astrocyte subclusters, and 4 neuroepithelial subclusters (Figure 6B). We wondered whether serum treatment could affect the cell fate trajectories of brain organoids. The control and serum‐treated brain organoids from both BO2 and BO3 showed similar trajectories with all cell subclusters and comparable cell population ratio for each subclusters (Figure 6B), indicating serum treatment does not change cell differentiation status in brain organoids. In addition, three major cell types were identified in brain organoids, including neurons, astrocytes, and neuroepithelial cells (Figure 6C). The neuronal population was the largest cell population detected in the organoids and was slightly reduced in serum‐treated brain organoids, while the astrocyte population was slightly increased in serum‐treated brain organoids (Figure 6C). Because neurons and astrocytes are highly associated with the pathology of AD, we focused on these two cell types for further analysis.

The number of differentially expressed genes (DEGs) that were up‐ or down‐regulated by serum treatment in neurons and neuronal subclusters are shown in Figure 6D. To find out what cellular functions of neurons were affected by serum treatment, DEGs were subjected to Gene Set Enrichment Analysis (GSEA) against Gene Ontology (GO) database. The GSEA results revealed that mitochondrial functions, like oxidative phosphorylation, electron transport chain and mitochondrion organization, were significantly enriched with positive normalized enrichment score (NES) in neurons of serum‐treated brain organoids (Figure 6E), consistent with published AD patient scRNA‐seq results showing abnormal mitochondrial functions in neurons of AD patient cortex.^[^ ^23^ ^]^ More importantly, multiple synaptic functions, such as synapse organization, synapse assembly, and synaptic signaling, were significantly enriched with negative NES values in neurons from serum‐treated brain organoids (Figure 6E). This result indicates that synaptic function and neural activity were reduced in neurons by serum treatment. Reduced synaptic function in neurons was observed in both serum‐treated brain organoids and AD patient cortex as revealed by direct comparison of GSEA analysis of neuronal DEGs in brain organoids and that in AD patient cortex published by Grubman et al.^[^ ^23^ ^]^ (Figure 6F). This result indicates that serum‐exposed brain organoids could recapitulate reduced synaptic function of neurons in AD patients. In addition, many synaptic genes were identified as DEGs in neurons of serum‐exposed brain organoids, the majority of which were down‐regulated by serum treatment, such as Cannabinoid receptor 1 (CNR1), GABA receptors (GABRB1 and GABRG2), glutamate receptors (GRIA1 and GRIA2), Leucine‐rich repeat‐containing protein 7 (LRRC7), and leucine‐rich repeat transmembrane neuronal proteins (LRRTM2 and LRRTM4) (Figure 6G). Many of these abnormally regulated synaptic genes were also identified as DEGs in AD patient cortex (Figure 6G). These results are consistent with our observation of synaptic loss and reduced neural activity in serum‐treated brain organoids (Figure 4) and provide a molecular explanation for the observation.

### 2.7 Single Cell Transcriptomic Analysis Reveals AD Features in Astrocytes of Serum‐Exposed Brain Organoids

**Figure 7:**
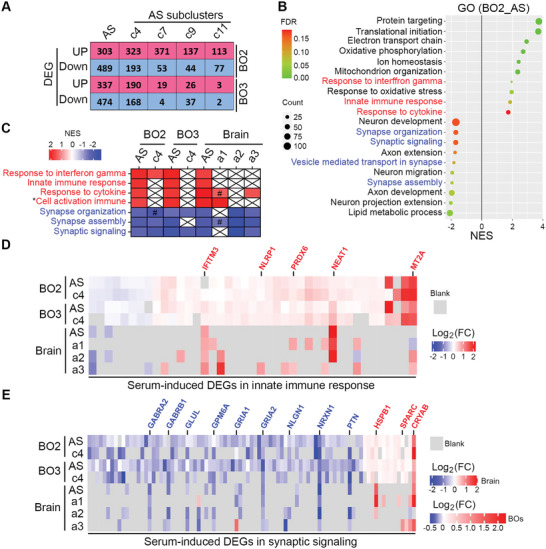
Serum exposure induces AD features in astrocytes of brain organoids as revealed by scRNA‐seq. A) The DEG numbers of astrocytes (AS) and AS subclusters that was up‐ or down‐regulated in serum‐treated BOs. B) GSEA result using DEGs of AS in BO2. FDR < 0.05. C) Multiple GO categories related to immune response and synaptic function are shown in astrocytes (AS) and AS subclusters of serum‐treated BOs and AD cortex published by Grubman et al. Colored by NES value for each category. *p*‐*v*alue < 0.05 except labeled with #. #: *p*‐value larger than 0.05 and less than 0.1. * indicates full name: cell activation involved in immune response. D) DEGs of AS involved in immune response from both serum‐treated BOs and AD cortex published by Grubman et al. Colored by Log2(FC) values. E) DEGs of AS involved in synaptic functions from both serum‐treated BOs (*p*‐value < 0.05) and AD cortex (FDR<0.05) published by Grubman et al. Colored by Log2(FC) values. FC: fold change.

In order to evaluate the effects of serum exposure on astrocytes, we compared gene expression levels in astrocytes isolated from serum‐treated brain organoids and control organoids. The number of DEGs that were up‐ or down‐ regulated by serum exposure in astrocytes and astrocyte subclusters from both BO2 and BO3 are shown in **Figure** **7**A. To identify biological processes that were affected by serum treatment in astrocytes, DEGs were subjected to GSEA analysis against GO database. Multiple GO terms related to immune responses, such as innate immune response, response to interferon gamma, and response to cytokines, were significantly enriched with positive NES in astrocytes of serum‐treated brain organoids (Figure 7B). The enhanced immune response was observed in astrocytes of both serum‐treated brain organoids and AD patient cortex as revealed by direct comparison of GSEA analysis of astrocytic DEGs in brain organoids and that in AD patient cortex published by Grubman et al.^[^ ^23^ ^]^ (Figure 7C). This result indicates that serum‐treated brain organoids could recapitulate enhanced immune response in astrocytes of AD patients. Many genes involved in immune response were identified as DEGs in astrocytes of serum‐exposed brain organoids, the majority of which showed increased expression under serum exposure (Figure 7D). In addition, many genes involved in immune response were identified as DEGs with increased abundance in astrocytes of both serum‐treated brain organoids and AD patient cortex, such as metallothionein‐2A (MT2A), nuclear paraspeckles assembly transcript 1 (NEAT1), peroxiredoxin‐6 (PRDX6), NACHT, LRR, and PYD domains‐containing protein 1 (NLRP1), and interferon‐induced transmembrane protein 3 (IFITM3) (Figure 7D). These results provide evidence supporting that enhanced immune response in AD patient brains could be induced by serum exposure.

Moreover, GSEA analysis revealed that multiple synaptic functions, like synapse organization, synapse assembly, and synaptic signaling, were significantly enriched with negative NES in astrocytes of serum‐treated brain organoids (Figure 7B), indicating that serum exposure could reduce synapse‐related functions in astrocytes of brain organoids. The reduced synaptic functions were observed in astrocytes of serum‐treated brain organoids (both BO2 and BO3) and AD patient cortex as revealed by direct comparison of GSEA analysis of astrocytic DEGs in brain organoids and that in AD patient cortex published by Grubman et al.^[^ ^23^ ^]^ (Figure 7C). This result indicates that serum‐treated brain organoids could recapitulate reduced synaptic function in AD patients. Many synaptic genes were identified as DEGs in astrocytes of serum‐exposed brain organoids, the majority of which showed decreased expression after serum treatment (Figure 7E). In addition, many synaptic genes with reduced expression were identified as DEGs in astrocytes of both brain organoids and AD patient brains, such as GABA receptors (GABRA2 and GABRB1), glutamate receptors (GRIA1 and GRIA2), Glutamine synthetase (GLUL), neuronal membrane glycoprotein M6‐a (GPM6A), neuroligin‐1 (NLGN1), neurexin genes (NRXN1), and pleiotrophin (PTN) (Figure 7E). These results revealed reduced synaptic functions in astrocytes of serum‐treated brain organoids at molecular level, consistent with our observation of synaptic loss and reduced neural activity in serum‐exposed brain organoids (Figure 4).

### 2.8 Serum Exposure Induces Activator of Apoptosis Harakiri (HRK) Gene and Apoptosis in Brain Organoids

As mentioned above, several mitochondrial functions were significantly enriched in serum‐treated brain organoids (Figure 6E,7B), suggesting mitochondrial dysfunction in serum‐treated brain organoids. Because mitochondrial dysfunction could cause cellular apoptosis, and the expression of HRK, a mitochondrial gene that induces apoptosis, was altered in serum‐treated brain organoids in our single‐cell RNA sequencing analysis. We validated the expression of HRK in control and serum‐treated brain organoids. Increased mRNA level of HRK in serum‐treated brain organoids was detected by real‐time PCR analysis (Figure S1A, Supporting Information). We next determined whether serum exposure could induce apoptosis in brain organoids. Immunoblotting for cleaved‐caspase 3, a marker for apoptosis, revealed that serum exposure increased the level of cleaved‐caspase 3 in brain organoids (Figure S1B,C, Supporting Information), indicating increased apoptosis in serum‐treated brain organoids.

## 3 Discussion

In this study, we modeled sAD using hiPSC‐derived brain organoids under serum exposure condition, mimicking serum exposure resulted from BBB leakage in brains of aged individuals^[^ ^12^, ^13^ ^]^. This serum‐exposed brain organoid model well recapitulated AD pathological features, including A*β*‐like pathology, elevated level of p‐Tau, synaptic loss, and impaired neural network. Moreover, we showed that serum exposure could elevate A*β* and p‐Tau levels through enhancing BACE and GSK3*α*/*β* expression/activity, respectively.

There is a high failure rate in drug development for AD.^[^ ^3a,b^ ^]^ The majority of the anti‐AD drug candidates are focused on targeting a single AD pathology like A*β*.^[^ ^24^ ^]^ Increasing evidences support the idea that BBB leakage plays critical roles in AD pathogenesis.^[^ ^13^, ^18^, ^25^ ^]^ Recently, an elegant 3D microfluidic model with human neural cell cultures was developed that could recapitulate key aspects of BBB dysfunction in AD.^[^ ^15a^ ^]^ A hiPSC‐based 3D model has provided novel insights into pericyte ApoE4‐induced cerebral amyloid angiopathy.^[^ ^13c^ ^]^ In this study, we demonstrated that serum exposure could induce multiple AD pathological features independently. Therefore, more effective strategies for AD therapeutic development may need to target more upstream events, such as BBB leakage, or target multiple downstream pathological phenotypes in parallel. Reducing serum exposure in AD patient brains could be a promising way to block AD pathological progression, because it could impact multiple AD phenotypes. Identifying functional serum components that could induce AD pathologies in the future may provide novel targets for developing effective therapeutic strategies for AD.

While hiPSC‐derived cells provide an excellent model to study the genetic risk factors for AD by manipulating the genetics of iPSCs, our study demonstrated a way to define the effects of non‐genetic risk factor(s) for AD by exposing brain organoids to non‐genetic risk factors. Because AD is an age‐related neurodegenerative disease, our study using brain organoids exposed to serum that could result from BBB leakage in brains of aged individuals^[^ ^12^, ^13^ ^]^ provides a way to involve age‐related events in AD modeling, thus allowing opportunities for us to detect age‐related AD pathological features and identify the underlying mechanisms.

Human iPSCs‐derived brain organoids have been widely used to model AD, including both fAD^[^ ^11b, e^ ^]^ and sAD,^[^ ^11d,g^ ^]^ which have provided great insights into AD pathogenesis caused by genetic factors. In this study, we found that serum exposure could induce elevated levels of A*β* and p‐Tau through increasing the expression and/or activity of BACE and GSK3*α*/*β*, respectively. In addition, we were able to recapitulate another AD pathological feature, the synaptic loss, in serum‐exposed brain organoids. We found that serum exposure led to perturbed expression of synaptic genes in neurons and astrocytes as revealed by single‐cell transcriptomic analysis, providing a plausible explanation for serum‐induced synaptic loss. Blocking either A*β* production or reducing p‐Tau level using respective inhibitors could not rescue serum exposure‐induced synaptic loss, suggesting that serum exposure could induce synaptic loss independently of A*β* or p‐Tau level, which may help us to uncover unexplored mechanisms underlying AD pathogenesis in the future.

In summary, this study provides a model for sAD using serum‐exposed hiPSC‐derived brain organoids, which could recapitulate multiple AD features, including elevated A*β* and p‐Tau levels synaptic loss, and impaired neural network. Single cell‐RNA seq analysis revealed down‐regulated synaptic function in both neurons and astrocytes in serum exposed brain organoids, providing a mechanistic basis for serum exposure‐induced AD‐like pathologies in the organoid model. The sAD brain organoid model established in this study provides a platform for both mechanistic study and therapeutic development for AD. This model in combination with a brain organoid system with both cerebrovasculature and blood stream that we envision could be developed in the future will provide further insights into the roles of BBB breakdown in AD pathogenesis.

## 4 Experimental Section

### Human Brain Tissues and Serums

Frozen brain tissues from post‐mortem AD patients and age‐comparable healthy control individuals were obtained Banner Sun Health Research Institute. Serum 1 and Serum 2 were isolated from anonymous blood specimens from individual donors, which were collected from the Michael Amini Transfusion Medicine Center of City of Hope, by centrifugation with 2000 × *g* at 4 °C followed by passing a 0.2 µm filter. Serum 3 was purchased from Sigma (H6914). The evaluation from Institutional Review Board determined these anonymous brain tissues and serums do not meet the definition of human subject research.

### hiPSC Derivation

hiPSCs including iPSC1, iPSC2 and iPSC5,were reprogrammed from human fibroblasts, which were purchased from Coriell and reprogrammed as described previously through episomal reprogramming using episomal plasmids expressing OCT4, SOX2, L‐MYC, KLF4, shp53, and EBNA1 (Addgene plasmids pCXLE‐hSK, pCXLE‐hUL, pCXLE‐hOCT3/4‐shp53‐F, and pCXWB‐EBNA1)^[^ ^5^, ^10^, ^26^ ^]^. Specifically, human fibroblast cells were electroporated with the reprogramming factors using 4D Nucleofector (Lonza) and seeded into 6‐well plates coated with 1:100 diluted Matrigel (Corning) and maintained in E8 medium (Invitrogen). The iPSC3, iPSC4 and iPSC6 hiPSCs were generated by the UCI ADRC Induced Pluripotent Stem Cell Core from UCI Alzheimer's Disease Research Center (ADRC) subject fibroblasts under approved IRB and SCRO protocols. Non‐integrating Sendai virus was used for iPSC3, iPSC4 and iPSC6 reprogramming (Cytotune 2.0). All iPSCs were confirmed to be karyotypically normal and pluripotent via teratoma formation or in vitro trilineage differentiation. In addition, all lines are routinely tested to confirm lack of mycoplasma contamination. hiPSCs were maintained at 37 °C in Matrigel‐coated 6‐well plates with daily medium change and passaged every 3–4 days using 0.5 mm EDTA treatment and manual dissociation. All the hiPSC lines are listed in Table S1, Supporting Information.

### Generation of Brain Organoids Using hiPSCs

The hiPSCs‐brain organoids were generated by the authors' published protocol,^[^ ^10e^ ^]^ as shown in Figure 1A. In order to generate brain organoids with good reproducibility and homogeneity, a screening was performed from Day 20 to Day 40 and only the organoids with the typical phenotype as showed in Figure 1A (Day 20, Day 40), which were mainly composed of multiple neural tube‐like rosettes, were kept for further culture.

### Serum and Compounds Treatment of Brain Organoids

The brain organoids were treated without (control) or with 10% human serum for ≈12 days and were harvested between Day 90 and Day 110 for experiments. For compound treatment, the brain organoids were treated 10% serum combined with vehicle (DMSO) control, 5 µm BACE Inhibitor IV (Sigma), or 1 µm GSK3 inhibitor CHIR99021 for 12 days and then were harvested between Day 90 and Day 110 for experiments.

### Immunostaining of Brain Organoids

The brain organoids were washed with phosphate‐buffered saline (PBS) and fixed with 4% paraformaldehyde (PFA) for 1–2 h at 4 °C, followed by 30% sucrose incubation overnight at 4 °C. Then the brain organoids were embedded in OCT and sectioned at a thickness of 20 µm using Leica CM3050S. The brain organoid sections were washed with PBS before incubation with 3% donkey serum in PBS with 0.1% Triton X‐100 for 1 h at room temperature (RT). Then the sections were incubated overnight at 4 °C with the primary antibodies diluted in 3% donkey serum in PBS with 0.01% Triton X‐100, followed by wash in PBS with 0.01% Triton X‐100 and incubation with secondary antibodies. Finally, the sections were counterstained with DAPI before mounting. Images were obtained with a Carl Zeiss LSM700 confocal microscope. The antibodies are listed in Table S2, Supporting Information.

### Immunostaining of Human Brain Tissues

The frozen brain cortex tissues of AD patients and healthy controls were fixed with 4% PFA overnight at 4 °C, followed by 30% sucrose incubation overnight at 4 °C. Then the brain tissues were embedded in OCT and sectioned at a thickness of 20 µm using Leica CM3050S. Antigen retrieval was performed in citrate buffer, pH 6.0 (Sigma), heated by microwave, and wash the sections with water after cool at RT. Because human brain tissues showed high autofluorescence at FITC and Cy3 channels, a quenching procedure was used to reduce background autofluorescence, which is similar to that described by Sun et al.^[^ ^27^ ^]^ with some modifications. The brain sections were incubated with 0.3% KMnO_4_ (w/v) for 5 min, washed in water, then treated with a solution of 1% K_2_S_2_O_5_ and 1% oxalic acid until the brown color was removed from the tissues (≈20 s). Washed the sections in water and repeated the quenching procedure once. After wash with water, the brain tissue sections were ready for immunostaining.

### Soluble and Insoluble Fraction of Brain Organoids

Brain organoids were lysed in PBS buffer containing 2 mm Na_3_VO_4_, 2 mm NaF and protease inhibitor cocktail set I (Calbiochem), followed by passing a syringe with G27 needle 40 times. After centrifugation with 15 000 × *g* for 10 min at 4 °C, the supernatants were the soluble fraction. The pellets were dissolved in sample loading buffer with 2‐mercaptoethanol and boiled for 10 min. After centrifugation with 15 000 × *g* for 10 min at 4 °C, the supernatants were the insoluble fraction. Protein concentrations were measured by Bradford Assay (Bio‐Rad).

### Western Blot

Brain organoids were lysed in RIPA buffer containing 2 mm Na_3_VO_4_, 2 mm NaF and protease inhibitor cocktail set I (Calbiochem), followed by sonication and centrifugation with 15 000 × *g* for 10 min at 4 °C. Protein concentrations were measured by Bradford Assay (Bio‐Rad). Proteins lysates were loaded for SDS‐PAGE and then were transferred to PVDF membranes, which were then blocked in 5% non‐fat milk in PBST for 1 h at RT followed by primary antibody incubation overnight at 4 °C in blocking buffer. Washed in PBST and incubated with secondary antibody in PBST for 1 h at RT. The membranes were washed in PBST and then were developed using ECL kit (GE Healthcare) and imaged using ChemiDoc Imaging System (Bio‐Rad). The antibodies are listed in Table S2, Supporting Information.

### Real‐Time PCR

Total RNA was extracted from control and serum‐treated brain organoids using TRIzol Reagent (Thermo Scientific) according to the manual. Then, 2.5 µg total RNA was used to synthesize cDNA using Tetro cDNA Synthesis Kit (Bioline) using random hexamer according to the manual. Real‐time PCR was performed using SYBR Green Master Mix (Thermo Scientific) on the Step One Plus Real‐Time PCR Instrument (Applied Biosystems). The Primers for real‐time PCR are listed in Table S3, Supporting Information. Diluted cDNA was used for reference gene 18s rRNA. Each reaction was run in triplicate. Data were analyzed using ΔΔCt method and normalized to control group in each run.

### A*β*_1‐40_ ELISA

The A*β*_1‐40_ levels in brain organoid lysates was detected using Human Amyloid beta (aa1‐40) Quantikine ELISA Kit (R&D) according to the manufacturer manual. And the A*β*_1‐40_ amount was normalized to 1 mg protein lysates.

### Transmission Electron Microscopy

Brain organoids were fixed with 2.5% glutaraldehyte, 0.1 m Cacodylate buffer (Na(CH_3_)_2_AsO_2_•3H_2_O), pH7.2, at 4 °C. Standard sample preparation for TEM was followed including post‐fixation with osmium tetroxide, serial dehydration with ethanol, and embedment in Eponate.^[^ ^28^ ^]^ Ultra‐thin sections (≈70 nm thick) were acquired by ultramicrotomy, post‐stained, and examined on the FEI Tecnai 12 transmission electron microscope equipped with a Gatan OneView CMOS camera.

### Calcium Imaging

Brain organoids at around day 90 of differentiation were dissociated and seeded on matrigel coated Ibidi µ‐slide 8‐well‐chamber slides and allowed to grow for one week until calcium imaging was performed following previous procedure.^[^ ^10e^ ^]^ Brain organoids were rinsed in artificial‐cerebrospinal fluid (ACSF) (124 mm NaCl, 2.5 mm KCl, 26 mm NaHCO_3_, 1 mm MgCl_2_, 2 mm CaCl_2_, 1.25 mm NaH_2_PO_4_ and 10 mm d‐glucose solution) at 37°C for 10 min and then incubated in fresh 95% O_2_ oxygenated ACSF containing 2 µm Fluo‐4 AM (Invitrogen) for 20 min. Subsequently, brain organoid tissue was visualized using a Zeiss Axio Observer Z1 microscope for serial time lapse imaging. Time lapse imaging was acquired at 10x magnification at 16 frames per second for 5 min using a Hamamatsu EMCCD model C9100‐13. Images were captured and processed using ZEN software (Carl Zeiss) and quantification was performed using Image‐Pro Premier 9.1 (Media Cybernetics). Fluorescence intensity change over time is defined as Δ*F/F* = (*F − F_o_*)/*F_o_*, where *F* is the fluorescence intensity at any time point, and *F_o_* is the baseline fluorescence intensity averaged across the whole movie for each cell. 10% serum was added to culture medium once the brain organoids were attached. Ca^2+^ imaging was performed one week after serum treatment. Medium without serum was used as control.

### Microelectrode Arrays

Brain organoids at around day 90 of differentiation were dissociated and seeded into 12‐well transparent MEA plates at three brain organoids per well (Cytoview MEA plate, Axion Biosystems). Brain organoid MEA processing and recording essentially followed previous procedure.^[^ ^10e^ ^]^ Briefly, brain organoids were fed with BrainPhys media (STEMMCELL Tech), including 1xB27 (Gibco), 1xN2 (Gibco), 20 ng mL^–1^ GDNF (R&D), 20 ng mL^–1^ BDNF (R&D), 500 µg mL^–1^ db‐cAMP (Sigma‐Aldrich), 1x Glutamax (Gibco), and 1x NEAA (Gibco), once a week and measurements were taken before medium change. MEA recordings were performed in a Maestro MEA system and AxIS software (Axion Biosystems) using a bandwidth with a filter for 10Hz to 2.5 kHz cutoff frequencies. Spike detection was performed using an adaptive threshold set to 5.5 times the standard deviation of the estimated noise on each electrode. For recordings, following a 5 min resting time in the Maestro Instrument, each plate was recorded for 10 min to calculate the spike rate per well. When a recording of 5 spikes over the length of 1 min (5 spikes per min) was obtained, the electrode was considered active. Individual electrode bursts were identified using an adaptive Poisson surprise algorithm, while network bursts were identified for each well using a non‐adaptive algorithm requiring a minimum of 10 spikes with a maximum inter spike interval of 100 ms. Multielectrode data analysis was performed using the Axion Biosystems NeuralMetrics Tool. Synchrony index were calculated by NeuralMetric Tool with synchrony window set as 20 ms. 10% serum was added to culture medium once the brain organoids were attached. MEA recoding was started one week after serum treatment. Medium without serum was used as control.

### Single Cell RNA Sequencing (scRNA‐seq) of Brain Organoids

Brain organodis treated without (control) or with serum 1 for ≈12 days were dissociated to single cells using the Neural Tissue Dissociation Kit (P) (Miltenyi Biotec) according to the manufacture's instruction. The cells were captured per sample on a 10x Chromium device using a 10X V3 Single Cell 3’ Solution kit (10xGenomics, Chromium Single Cell 3’ Regent kit V3 Chemistry, Cat. PN‐1000092). All protocols were performed following the manufacture's instruction. Final sequencing libraries were analyzed on a High Sensitivity DNA Chip (Agilent, Cat 5067‐4626) to determine the library size, and library concentration was determined with a Qubit high Sensitivity DNA assay Kit (Thermo, Cat. Q32854). The libraries were sequenced with the paired end setting of 28 cycles of read1, 101 cycles of R2 and 8 cycles of index read on Illumina NovaSeq 6000 platform at Tgen.

### Mapping Single Cell Reads to Human Genome

Raw sequencing data were processed using the 10x Genomics’ Cell Ranger pipeline (version 3.1.0) to generate FASTQ files and aligned to hg19 genome to generate gene expression count for transcriptome version hg19‐1.2.0 from 10x Genomics.

### Preprocessing of scRNA‐seq Data

The number of genes was quantified with expression greater than zero in each cell and then the cells expressing fewer than 200 genes in each sample was removed. To uphold against undetected doublets, the outlier cells expressing >9000 genes were removed. The cells with more than 25% mitochondria reads for all samples were also removed. There are 26 631 cells left including 9338 cells from BO2_control, 5412 cells from BO2_serum, 6535 from BO3_control, and 5346 cells from BO3_serum. The datasets were normalized by a global‐scaling method “LogNormalize” followed by a linear transformation (scaling), and all the scaled datasets were integrated into one. The Seurat R package^[^ ^29^ ^]^ was used to carry out data normalization, integration and scaling, as well as downstream dimensionality reduction, clustering, UMAP plot overlaying, and differential expression.

### Dimensionality Reduction and Clustering

The authors concentrated on the most informative genes for use in dimensionality reduction. To achieve these, the highly variable features were selected through calculating a subset of features that exhibit high cell‐to‐cell variation in the dataset. Dataset has more than 2000 feature were selected. These datasets were subjected to principal component analysis (PCA) and then, the top 14 PCs (principal components) were selected for subsequent clustering and visualization.

Single cell samples were clustered in an unsupervised manner based on their expression by first constructing a shared nearest neighbor graph based on k‐nearest neighbors (KNN) calculated from the first 14 PCs of the scaled data (*k* = 20). The number of clusters was then determined using a modularity function optimizer based on the Louvain algorithm (resolution, 0.5).

To reduce dimensionality further, the Uniform Manifold Approximation and Projection (UMAP) was performed based on the top 14 PCs and plotted this embedding in 2D space (number of neighbors, 30; number of components, 2).

### Annotation of Cell Types

SingleR^[^ ^30^ ^]^ was used to characterize cells types in clusters based on reference transcriptomic datasets of pure cell types. To annotated cells, a reference database was built using Human Primary Cell Atlas.^[^ ^31^ ^]^ ScoreHeatmap was generated and DeltaDistribution plot were generated to inspect the confidence of the annotated labels across the dataset.

### Identification of Differentially Expressed Genes

In order to identify the differentially expressed genes (DEGs) between serum‐treated cells and control cells, the Wilcoxon Rank Sum test is used to identify gene expression changes between the two groups of cells. The DEGs were defined as having FDR (adjusted *p*‐value) less than 0.05 and absolute Log _2_(fold change, serum vs control) > 0.1.

### Functional Enrichment Analysis

The DEGs of each cell type or subcluster were used for gene set enrichment analysis (GSEA)^[^ ^32^ ^]^ based on the Molecular Signatures Database (MSigDB).^[^ ^33^ ^]^ Bobble plots showed expression of enriched pathways in each cell clusters. Enrichment score indicated the degree to which a gene set is overrepresented at a given gene set database.

### Comparison with snRNA‐seq Data of AD Patient Brains Published by Grubman et al

DEG results of neurons, astrocytes and their subclusters between “Alzheimer's disease” and “healthy control” were downloaded from Grubman et al.^[^ ^23^ ^]^ with absolute Log_2_ (fold change) > 0.5 and FDR < 0.05. Then, the DEGs in each cell type and subclusters were used to perform the GSEA analysis based on the Molecular Signatures Database (MSigDB). The categories related to immune response, Alzheimer's disease pathway, mitochondrial functions and synaptic functions were compared to the authors' GSEA results of brain organoids.

### Quantification and Statistical Analysis

Statistical significance was analyzed using Graphpad Prism Version 8.1.1 by unpaired two‐tailed *t*‐test. For all test, *p*‐values were presented as *p < 0.05, **p < 0.01, ***p < 0.001, and ****p < 0.0001. Error bar stands for SD. Statistical details of each experiment can be found in the figure legends.

## Conflict of Interest

T.G.B. has contract research with Avid Radiopharmaceuticals. E.M.R. is a scientific advisor to Alkahest, Alzheon, Aural Analytics, Denali, Green Valley, MagQ, Takeda and United Neuroscience. E.M.R. is an advisor to Roche/Roche Diagnostics and Cerveaux (expenses only). E.M.R. is co‐founder and shareholder of AlzPath, a new company which aims to advance the role of blood‐based biomarkers for Alzheimer's disease research, drug development and care. M.B.J. is a co‐inventor of patent WO/2018/160496 related to differentiation of human pluripotent stem cells into microglia. D.M.H. is as an inventor on a patent licensed by Washington University to C2N Diagnostics on the therapeutic use of anti‐tau antibodies. D.M.H. co‐founded and is on the scientific advisory board of C2N Diagnostics. C2N Diagnostics has licensed certain anti‐tau antibodies to AbbVie for therapeutic development. D.M.H. is on the scientific advisory board of Denali and consults for Genentech, Merck, and Cajal Neuroscience.

## Author Contributions

X.C., Y.S., and D.M.H. designed and Y.S. directed this study. X.C. developed the brain organoid protocol, generated the brain organoids, and performed the majority experiments. G.S. did the calcium imaging and MEA. E.T. generated and characterized iPSC1, iPSC2, and iPSC5. M.Z. performed ELISA. H.D. and M.B.J. generated and characterized iPSC3, iPSC4 and iPSC6. T.B. and E.M.R. provided healthy control and AD patient brain tissues and helped interpretation of findings. X.C. and Y. S. prepared the manuscript with input from all other authors.

## Supporting information

Supporting Information

## Data Availability

The single‐cell RNA sequencing data published by Grubman et al. are available from the Gene Expression Omnibus (GEO) under the accession number GSE138852. The scRNA‐seq data of brain organoids generated in this study have been deposited under accession number GSE164089.
