# Supplementary material for: Modeling Sporadic Alzheimer's Disease in Human Brain Organoids under Serum Exposure
Source: Adv Sci (Weinh). 2021 Aug 2;8(18):2101462. doi: 10.1002/advs.202101462 (PMC8456220; doi:10.1002/advs.202101462)
Supplement: Supplementary file 1 — Supporting Information [file ADVS-8-2101462-s001.pdf]

© 2021 Wiley-VCH GmbH

ADVANCED SCIENCE Open Access

Supporting Information

for *Adv. Sci.,* DOI: 10.1002/advs.202101462

Modeling sporadic Alzheimer's disease in human brain organoids under serum exposure

*Xianwei Chen, Guoqiang Sun, E Tian, Mingzi Zhang, Hayk Davtyan, Thomas Beach, Eric M. Reiman, Mathew Blurton-Jones, David Holtzman and Yanhong Shi**

Supporting Information

Modeling sporadic Alzheimer's disease in human brain organoids under serum exposure

*Xianwei Chen, Guoqiang Sun, E Tian, Mingzi Zhang, Hayk Davtyan, Thomas Beach, Eric M. Reiman, Mathew Blurton-Jones, David Holtzman, Yanhong Shi**

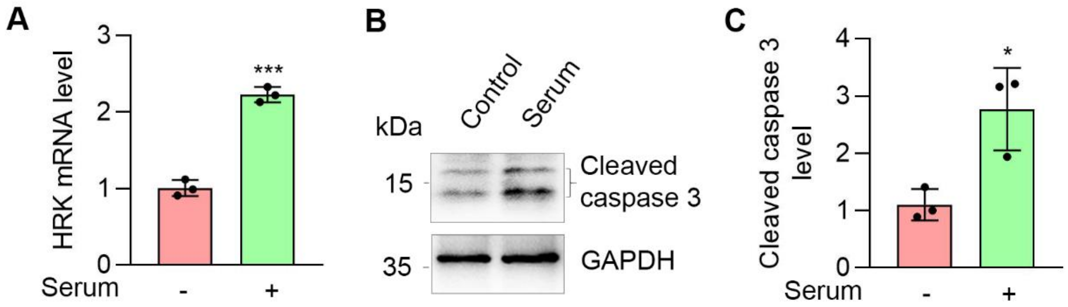

Figure S1. Serum exposure induces HRK gene and apoptosis in brain organoids. A) HRK mRNA levels in control and serum-treated BO5 detected by real-time PCR, and were normalized to 18s rRNA. Total RNA was extracted from pooled 9-10 individual BOs. Error bars are SD of the mean; n = 3 experimental repeats. ***p<0.001 by unpaired two-tailed t test. B) Western blot of cleaved caspase 3 and GAPDH levels in control and serum-treated BO1. Each lysate was from pooled 3-5 individual BOs. Quantification of cleaved caspase is shown in panel (C) and normalized to GAPDH. Error bars are SD of the mean; n = 3 experimental repeats. *p<0.05 by unpaired two-tailed t test.

Table S1. The hiPSC lines for generating brain organoids.

Brain organoids	hiPSCs	Parental cells	Age	Gender	HC[a)]/AD[b)]
BO1	iPSC1	Fibroblast	60	Female	AD
BO2	iPSC2	Fibroblast	71	Male	HC
BO3	iPSC3	Fibroblast	81	Female	AD
BO4	iPSC4	Fibroblast	67	Female	HC
BO5	iPSC5	Fibroblast	87	Male	HC
BO6	iPSC6	Fibroblast	72	Female	AD

a) healthy control; b) Alzheimer's disease.

Table S2. The list of antibodies.

Antibodies	Vender	Catalog number
Goat polyclonal anti-SOX2	R&D	AF2018
mouse monoclonal anti-TUJ1	Covance	PRB-435P
Rat monoclonal anti-CTIP2	abcam	Ab18465
Rabbit monoclonal anti-TBR2	abcam	ab216870
Mouse monoclonal anti-SATB2	abcam	ab51502
Chicken poloclonal anti-MAP2	abcam	ab5392
Mouse monoclonal anti-GFAP	Sigma	G3893
Rabbit monoclonal anti-Aβ (D54D2)	Cell Signaling	8243S
Mouse monoclonal anti-Aβ (6E10)	Biolegend	803004
Mouse monoclonal anti-p-Tau (AT8)	Invitrogen	MN1020
Mouse monoclonal anti-p-Tau (AT270)	Invitrogen	MN1050
Mouse monoclonal anti-GAPDH	Santa Cruz	SC-47724
Mouse monoclonal anti-Tau (TAU-5)	Millipore	577801
Rabbit monoclonal anti-BACE (D10E5)	Cell Signaling	5606S
Mouse monoclonal anti-BACE (61-3E7)	Santa Cruz	SC-33711
Rabbit monoclonal anti-p-GSK3α/β (Y216/Y279)	abcam	ab68476
Rabbit monoclonal anti-GSK3α/β	Cell Signaling	5676S
Rabbit polyclonal anti-SYN1	synaptic system	106 103
Rabbit polyclonal anti-cleaved caspase 3	Cell Signaling	9661S

Table S3. The list of primers for real-time PCR.

Primer name	Sequence
HRK forward	5'- CAGGCGGAACTTGTAGGAAC-3'
HRK reverse	5'- GGCCTTTCAAGCTCTGGG-3'
18s rRNA forward	5'-GAGACTCTGGCATGCTAACTAG-3'
18s rRNA reverse	5'- GGACATCTAAGGGCATCACAG-3'
